# Assessing the quality of risk assessment conducted for new psychiatry inpatients

**DOI:** 10.1192/bjo.2021.251

**Published:** 2021-06-18

**Authors:** Nikhita Handa

**Affiliations:** East Lancashire Hospitals NHS Trust

## Abstract

**Aims:**

An audit was conducted to assess if thorough risk assessments had been documented in electronic clinical record notes (ECR) clerking for new patients in two acute mental health wards. Risk assessment is a vital part of admission clerking and when done well it can prevent early incidents and aid the ward nursing team greatly. During induction, junior doctors are advised to document assessed risks when clerking a new patient. A screening of the risks on admission could help determine the levels of observations required to minimise the identified risks whilst the patient awaits their first ward review.

**Method:**

The NHS numbers for the 30 current inpatients across male and female acute psychiatric wards were gathered at the time of the audit (February – March 2020). Admission clerking was analysed for a clear statement of patient risk to self, others or property. Within these categories quantitative results were obtained on how often the risk of self-harm, self-neglect, absconding, vulnerability or aggression was documented. The term ‘risk’ was used for each patient on their ECR notes to search for risk assessments in all entries other than admission clerking.

**Result:**

12 out of the 30 patients had a junior doctor risk assessment documented in their clerking (40%). 14 patients had no mention of risk assessment on admission (47%) and their first formal risk assessment was documented only in their senior ward review. Of the 12 assessments completed in clerking; all assessed self harm/suicide risk and violent risk to others, 1 mentioned risk of absconding, 8 mentioned risk of illicit substance use and 8 mentioned vulnerability. It was unclear if the risks documented were based on current or historic presentation. Junior doctors were anonymously surveyed following this audit and reported they did not feel confident in how to document a risk assessment or whether to document negative findings.

**Conclusion:**

Clear documentation of risk assessment being performed was lacking in over half of junior doctor admission clerkings. When risks were assessed it was mainly violence/self harm risk documented not vulnerability and physical health risks. Based on these findings we have designed more comprehensive teaching on risk assessments and a template for how to complete a risk assessment. We feel the use of a template will ensure all elements of risk are clearly considered even if they are not present currently. This is being reaudited to assess if the changes have impacted the quality of risk assessment conducted.

